# The Effect of Lipopolysaccharide on Ischemic-Reperfusion Injury of Heart: A Double Hit Model of Myocardial Ischemia and Endotoxemia

**DOI:** 10.15171/jcvtr.2015.19

**Published:** 2015

**Authors:** Nader D. Nader, Mehrdad Asgeri, Sina Davari-Farid, Leili Pourafkari, Faraz Ahmadpour, Jahan Porhomayon, Hassan Javadzadeghan, Sohrab Negargar, Paul R. Knight

**Affiliations:** ^1^ Department of Anesthesiology, University at Buffalo, Buffalo, NY, USA; ^2^ Private Practicing Gastroenterologist, Cleveland Area, OH, USA; ^3^ Cardiovascular Research Center, Tabriz University of Medical Sciences, Tabriz, Iran; ^4^ Virox Inc., Oakville, Ontario, Canada

**Keywords:** Myocardium Ischemia, Reperfusion Injury, Tumor Necrosis Factor-Alpha, Endotoxemia

## Abstract

*Introduction:* Myocardial ischemia may coincide and interact with sepsis and inflammation. Our objective was to examine the effects of bacterial endotoxin on myocardial functions and cell injury during acute ischemia.

*Methods:* Rabbits were pretreated with incremental doses of *E. Coli* lipopolysaccharide (LPS) or normal saline. Myocardial ischemia was induced by 50-minute occlusion of left anterior descending artery. S-TNFaR was additionally used to block the effects LPS.

*Results:* Ventricular contractility as it was measured by dp/dt during systole decreased from 2445± 1298 to 1422 ± 944 mm Hg/s, *P* = .019. Isovolumetric relaxation time as an index of diastolic function was prolonged from 50±18 ms to 102± 64 ms following ischemia. Pretreatment with low concentrations of LPS (<1 μg) had no effect on dp/dt, while at higher concentrations it suppressed both contractility and prolonged IVRT. Cell injury as measured by cardiac troponin I level increased to 15.1± 3.2 ng/dL following ischemia and continued to rise with higher doses of LPS. While blocking TNFa did not improve the myocardial contractility after ischemia, it eliminated additional deleterious effects of LPS.

*Conclusion:* Lower doses of LPS had no deleterious effect on myocardial function, whereas higher doses of this endotoxin cause cardiac dysfunction and increased extent of injury.

## Introduction

Treatment of patients with sepsis in critical care settings often involves management of heart failure and myocardial dysfunction.^1^ Multi-organ failure is generally a sign of advanced septic shock and alters contraction and relaxation of cardiac myocytes.^2^ Levy and his colleagues have described metabolic responses to systemic sepsis and myocardial ischemia in an in vivo animal model.^3^ These authors have examined hibernation of the cardiac myocytes during sepsis. Fortunately, myocardial changes caused by systemic sepsis are transient in nature and complete recovery results with treating the underlying inflammatory response.

Occurrence of myocardial ischemia during sepsis is not uncommon. Increased oxygen consumption during sepsis may result in a full-blown acute coronary syndrome (demand ischemia) especially when it is superimposed to preexisting coronary disease. Irreversible myocardial infarction occurs when the heart muscle is deprived of oxygen and nutrients for prolonged durations of ischemia. Both ischemia and the reperfusion of are the causes of myocardial injury.^4^ One of the most important causes of these damages is free reactive species of oxygen; therefore many attempts to reduce the amount of injury by these radicals have been done.^5-7^

We have previously shown that recombinant TNFα has a dual effect on myocardial function after ischemia. While low dose TNFα improved the systolic function of the heart, the higher doses of this cytokine suppressed myocardial contractility and increased myoglobin leak from an ex vivo model of functioning heart following ischemia.^8^ We have also shown that TNFα is an important inflammatory cytokine, which is released in response to myocardial ischemia in human models.^9^ In this study the dose-response effects of the *E. Coli* endotoxin lipopolysaccharide (LPS) on ischemic hearts during reperfusion has been assessed. Intravenous injections of this endotoxin have been associated with dose response release of TNFα in a rabbit model of injury.^10^ We have tested the hypothesis that LPS is deleterious to the recovery of myocardium following an ischemia-reperfusion insult. The decision to use LPS rather than the rodent model of sepsis was to focus on Toll-like receptors superfamily (TLR-4) signaling in which TNFα plays a major role. Additionally, it is difficult to assess the dose-response relationship between the level of sepsis and myocardial injury in traditional models of sepsis (ie, cecal puncture ligation model).

## Materials and Methods

All experimental procedures and protocols used in this study were reviewed and approved by the Animal Use and Care Committee of the VA Medical Center at Buffalo and the care and handling of the animals were in accord with National Institutes of Health Guidelines. New Zealand White male rabbits, weighing 3.6 to 4.5 kg, were used for these experiments. The rationale for using rabbit model was due to its anatomical similarity to human coronary vessels and its larger size comparing to other rodent models.

### Experimental Design and Lipopolysaccharide Injection

Rabbits were housed for a period of one week before the experimentation. A total of 120 rabbits were used in these experiments; 11 rabbits deceased in the process without completing all the required steps and therefore were excluded from the final results. Deceased animals were then replaced until the desired number of animals in each group was achieved. On the day of the experiment, each rabbit was injected with a pre-calculated dose of LPS. The dose of LPS for each group was calculated on logarithmic increments and injected intravenously, prior to ischemia to stimulate TNFα production by the animal. Serum TNFα was measured to confirm the response to LPS. *E. Coli-*derived LPS was purchased from (Sigma Aldrich, St Louis, MO) and suspended in sterile normal saline solution 1 mg/mL and stored in the refrigerator until it was diluted and used. Sham-operated group was used as a control for surgical procedures. LPS administration was done 60 minutes prior to occlusion of left anterior descending artery.

In a separate group, in order to examine the role of TNFα in mediating the effects of LPS, soluble TNFα receptor (sTNFαR) etanercept (Enbrel^®^, Amgen Inc., Thousand Oaks, CA) 5 mcg in saline solution was injected subcutaneously 48 hours prior to surgery to inhibit TNFα. This dose (1.6 mcg/kg) was considered a “high dose regimen” and it was previously used in a rabbit model of arthritis to block the effects of TNFα.^11^ This soluble receptor was a fusion protein engineered to link the human gene for soluble TNF receptor 2 to the gene for the Fc component of human immunoglobulin G1. Soluble TNFαR was only used to in animals which received 100 µg of LPS since this dose was associated with peak TNFα response. Controls were injected with NS instead of LPS to examine the effect of sTNFαR on ischemic myocardium.

### Surgical Procedures, Anesthetic Techniques and Invasive Monitoring

Each rabbit was anesthetized with 25 mg/kg of ketamine intraperitoneally. Intubation of the trachea was done using a polyethylene tube No. 3.0 (Hudson RCI, Research Triangle Park, NC) following anesthesia, and mechanical ventilation was started using a tidal volume of 10 ml/kg at the rate of 25 breaths per minute. Anesthesia was maintained throughout the study by administering additional intra-peritoneal doses of ketamine 5 mg/kg, as needed and buprenorphine 0.05 mg/kg every 2 hours. Body temperature was monitored using a rectal probe and was kept between 36.5°C-37.5°C by placing a controlled heating plate under the animal during the experiment. Femoral arterial catheter was used for blood pressure measurements. Arterial blood pressures, electrocardiogram, and oxygen saturations were monitored throughout the experiment. Blood gas tensions and pH were monitored and were kept within the normal physiologic range (pH of 7.35-7.45 and PaCO2 between 30-40 torr). Experimental medications and maintenance IV fluid PlasmaLyte (Life Technology, Grand Island, NY) solutions at 25 ml/hour were administered via a venous line placed in the femoral vein.

Exposition of the heart through a median sternotomy and baseline readings were done at this time. Left ventricular pressure was measured by surgical advancement of a PE-15 (Med-Vet International, Libertyville, IL) catheter into the left ventricle and its global function parameters were calculated from pressure changes in one second (dp/dt) and isovolumetric relaxation time (IVRT). The segmental contractility of the myocardium was measured by sonomicrometer technology (Sonometrics^®^, London, Ontario) by attaching three crystal electrodes to the apex, anterior wall and posterior wall of the left ventricular base. Sensors were secured using a 6-0 prolene suture and segment shortening was measured using ultrasonic recordings. Hemodynamic parameters and cardiac function were measured continuously and recorded at baseline, upon induction of ischemia, immediately at the time of reperfusion and at the conclusion of 4 hours of reperfusion. Blood samples were collected from femoral arterial lines into tubes containing heparin powder 300 mg as anticoagulant (green top) and centrifuged at 2000 g to separate plasma from blood cells. Plasma samples were stored at -80°C until they were analyzed for TNFα and cardiac troponin I (cTnI) concentrations upon conclusion of experiments.

### Induction of Ischemia

**Figure 1:**
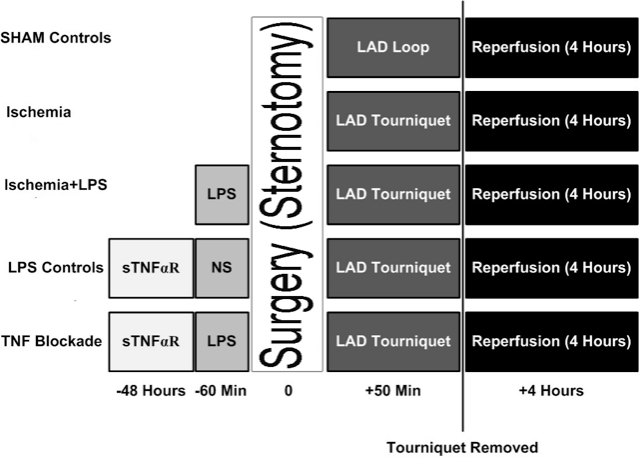
Timetable of the Treatments for the Experimental Groups.

After collection of baseline information, hearts from each ischemia group were treated with a 9-0 prolene suture loop around the left anterior descending artery, distal to the first diagonal branch. This level of occlusion produced a sublethal ischemia of the myocardium with ≤10% (11 out of 120 rabbits) mortality before completion of the experimental protocol. In the sham-operated group, the loop was not tightened allowing normal blood flow, whereas in the ischemia groups the loop was tightened to create an occlusion and induce myocardial ischemia. The occlusion of the coronary artery was maintained for 50 minutes (Figure 1). The ischemic region was reperfused then by loosening the suture loop for 4 additional hours. Ischemia was confirmed by epicardial cyanosis of the ischemic region, and reperfusion by a hyperemic response.

### Cell Injury and Local Inflammatory Response Assessment

Serum concentrations of cardiac troponin I (cTnI) were measured as a surrogate to the extent of myocardial injury. A commercially available ELISA kit was used to measure cTnI on polyvinyl microtiter 96-well plates (Life Diagnostics Inc., West Chester, PA). Duplicate assays were performed for each assay, and final colored plates were analyzed at 450 nm using an ELISA plate reader (Benchmark, Bio-RAD). Numeric readings were expressed in pg/ml of serum. TNFα levels in the serum were measured prior to the induction of ischemia to examine the cytokine response to LPS injection. For determination of serum TNFα concentrations, we similarly used an ELISA assay (Quantikine^®^, R&D Systems, Minneapolis, MN). The assays were performed on 96-well plates as described in the product guide material. The sensitivity of the test was 5 pg/mL and the range of measurement was 12.5-800 pg/mL.

The hearts were removed after 4 hours of reperfusion and the anterior wall of the left ventricle was minced and homogenized using a tissue homogenizer. Oxidative stress in ischemic tissue homogenates was analyzed by measuring tissue malondialdehyde (MDA) levels using a commercially available kit (Bioytech LPO-586TM Portland, OR). The base of this assay was based on the reaction of a chromogenic reagent, N-methyl-2-phenylindole, with MDA and 4-hydroxyalkenals at 45°C secondary to the reaction between one molecule of either MDA or 4-hydroxyalkenal with 2 molecules of the chromogenic reagent.

### Statistical Analysis

All data were maintained in a Microsoft Excel datasheet and were exported into a NCSS 2007 (Kaysville, Utah) database for statistical analyses. Differences of parametrical variables within and among the groups were assessed using one-way analysis of variance with repeated measurements. Bonferroni correction was used for post hoc analysis. Expressed data are presented as mean ± standard deviation. Null hypotheses were rejected at alpha error set at 0.05 or less.

## Results

### Ventricular Function

Baseline derivative of pressure over time (dP/dT) of the left ventricle was 2531 ± 912 mm Hg/s similar among all groups. After induction of ischemia, dP/dT decreased to 1422 ± 844 mm Hg/s from its respective values of 2445 ± 1018 mm Hg/s in SHAM-operated controls (*P* = .04) and remained suppressed throughout reperfusion period. Intravenous administration of low doses of LPS (<0.3 µg) prior to the induction of ischemia partially restored dP/dT. Additional increases in LPS dose were associated with further decreases in dP/dT (Table 1). The left ventricle was significantly improved when the ischemia plus 100 µg LPS group was treated with sTNFαR (781 ± 355 mm Hg/s vs. 1532 ± 854 mm Hg/s, *P *= .002) (Table 1).

**Table 1:** Physiological and Biochemical Variables for Various Treatment Groups

**Variables** **LPS (µg)**	**50 min Ischemia by applying LAD tourniquet**								**sTNFαR** **100 µg** **(n = 8)**
**None** **(n = 15)**	**0.1 µg** **(n = 8)**	**0.3 µg** **(n = 8)**	**1.0 µg** **(n = 8)**	**10 µg** **(n = 8)**	**30 µg** **(n = 8)**	**100 µg** **(n = 15)**	**300 µg** **(n = 8)**
Left Ventricle Contractile Function									
+dP/dT (mm Hg/s)	1422±844	1752±967	1733±1021	1499±1211	1321 ± 1201	1354±1380	1002±488	762±348	989±1004
SS (ISW) %	-5.3 ± 1.2	0.1 ± 1.5*	0.2 ± 1.6*	-2.5 ± 1.3	-3.4 ± 1.5	-4.8 ± 1.8	-5.9 ± 2.4	-6.8 ± 2.1	-5.1 ± 2.3
SS (NIW) %	15.1 ± 4.2	18.5 ± 5.1	16.8 ± 5.3	14.1 ± 5.2	11.1 ± 3.5	12.3 ± 5.3	6.4 ± 3.8 *	5.2 ± 2.0 *	5.9 ± 3.1
Left Ventricle Relaxation									
-dP/dT (mm Hg/s)	1022±944	1688±912	1622±895	1411±962	1123±745	1098±591	781±355	512±452	1032 ±854
IVRT (msec)	102 ± 64	86 ± 91	101 ± 84	109 ± 91	126 ± 95	116 ± 86	205 ± 101	311 ± 124	131 ± 96
Inflammatory Cytokines and myocardial Injury Biomarkers									
TNFα (pg/mL)	180 ± 56	345 ±79	434 ± 102	604 ± 362	893 ± 330	988±287	2350±1220	2403±1340	2421±1620
cTnI (ng/dL)	10.1 ± 3.2	10.2 ± 4.1	11.2 ± 7.6	18.9 ± 10.2	25.6 ± 13.1	31.2 ± 6.9*	37.5± 5.1†*	37.4 ± 8.1*	9.5 ± 3.6†
MDA (pg/mL)	180 ± 25	NM	NM	NM	NM	NM	650 ± 253 †*	2770±1240 *	285 ± 115†
MDA (pg/mL)	180 ± 25	NM	NM	NM	NM	NM	NM	NM	NM

All cardiac variables measured after 4 hours of reperfusion to examine the contractile and relaxation functions of the heart, as well as the marker of myocardial injury. Markers of myocardial contraction included derivatives of the left ventricle (LV) pressure over the derivatives of time during systole (+dP/dT) and the percent segment shortening (SS) of the anterior ischemic wall (ISW) and the posterior non-ischemic wall (NIW). In order to assess the relaxation of the LV, negative values of dP/dT were measured during diastole along with isovolumetric relaxation time in millisecond. Biomarkers of cellular injury and inflammation included serum concentrations of tumor necrosis factor alpha (TNFα, which are expressed in pg/mL, cardiac troponin I (cTnI) and malondialdehyde (MDA), a marker of lipid peroxidation, which is also expressed in pg/mL of tissue homogenate. Asterisks indicate a significant difference (*P *< .05), when a comparison was made to the ischemia controls (IS) and † indicates significant difference (*P *< .05), when a comparison was made between the groups treated with soluble receptor of TNFα to their normal saline-treated control (IS + LPS 100 µg).

Segment shortening was measured as a surrogate to regional contractile function of the myocardium and was examined in both ischemic and non-ischemic regions of the heart. In comparison to SHAM control with a shortening percentage of 12 ± 2.6%, the anterior ventricular wall (ischemic myocardium) segment shortening disappeared during systole and in fact there was a systolic lengthening of 5.3 ± 1.2% after ischemia was induced (Wall dyskinesia). Ventricular wall segment shortening in the ischemic region (ie, dysfunction) was improved with low doses of LPS treatment. The dyskinesia of the anterior ventricular wall (-5.3 ± 1.2%) due to ischemia improved to akinesia or hypokinesia of the ischemic wall at 0.1 ± 1.5% (*P *< .001) when ischemic hearts were treated with low doses of LPS (<0.3 µg) and reappeared when the dose of LPS exceeded 1 µg (Table 1).

Non-ischemic posterior segment shortening was measured to compare with the ischemic anterior wall segment shortening values. Posterior segment shortening increases with anterior wall ischemia in the control ischemia group (compensatory hyperkinesia). Lower doses of LPS (0.1 µg and 0.3 µg) increased the posterior wall segment shortening while higher LPS doses decrease the shortening percentage to below the levels achieved in the SHAM-operated control group. Soluble TNFαR treatment did not improve the posterior wall segment shortening in the ischemia plus 100 µg LPS group.

### Relaxation of Ventricular Muscle

Negative dP/dT and isovolumetric relaxation time (IVRT) were measured to assess ventricular relaxation. In the in vivo experiment, the baseline IVRT increased from 50 ± 18 milliseconds in the control sham to 102 ± 64 milliseconds in the ischemia control group. Negative dP/dT, did not change significantly with lower doses of LPS. LPS doses over 30 µg, impaired ventricular relaxation significantly, as shown by decreased dP/dT. Although adding sTNFαR alleviated ventricular pressure changes over the time, but in overall sTNFαR did not help ischemic myocardial function. Additionally, low doses of LPS (<30 µg) had no effect on the relaxation time of the ischemic hearts, while further increases of LPS dose prolonged the isovolumetric relaxation time (Table 1). IVRT was similar when the ischemia plus LPS 100 µg group was treated with sTNFαR (205 ± 101 milliseconds vs. 131 ± 96 milliseconds).

### Cytokine Response and Cellular Injury

Serum TNFα increased from 23 ± 1.5 pg/mL to 180 ± 56 pg/mL following myocardial ischemia (*P* < .01) when analyzed after reperfusion. The serum concentration of TNFα increased linearly in response to intravenous administration of LPS up to 30 µg and remained steady as the dose of LPS increased (R^2^ = 0.65, *P* < .01). sTNFαR administration had no effect on LPS-induced increases of serum TNFα following myocardial ischemia (Table 1). Serum cTnI concentrations, which were undetectable in SHAM injury group, increased following ischemia (10.1 ± 3.2 ng/dL) indicating structural myocardial damage. Serum cTnI levels continued to rise as increasing doses of LPS were administered, peaking at 37.5 ± 5.1 ng/dL following 100 µg of LPS. Further increases of the LPS dose did not increase the serum concentrations of cTnI (Table 1). Serum concentrations of cTnI and TNFα were directly correlated (R^2^ = 0.68, *P* < .001). Treating the rabbits with sTNFαR prevented the increases in cTnI in the ischemia plus 100 µg LPS group, thus the cTnI levels were close to that on the control ischemia group (Table 1).

Similarly, MDA concentrations increased from 23 ± 4.8 in SHAM controls to 180 ± 25 nmol/mL in ischemia controls. Administering 100 µg LPS to the ischemic animals further increased myocardial MDA levels to 650 ± 253 nmol/ml. Pretreating the animals with sTNFαR completely abolished LPS-induced increases of MDA levels (285 ± 115 nmol/ml) compared to controls (822 ± 119 nmol/ml). Following administration of 300 µg of LPS, the myocardial concentrations of MDA peaked at 2770 ± 1240 nmol/mL (Table 1).

## Discussion

This study made 2 new observations that might have important clinical implications. These findings were based on the experimental model of intravenous administration of LPS to animals with myocardial ischemia-reperfusion. Firstly, lower doses of LPS partially reversed the ischemia-induced impairment of myocardial contractility, as demonstrated by one grade improvement in fiber shortening of the myocardium from a dyskinetic to an akinetic state. However, with higher doses of LPS the left ventricular wall dyskinesia reappeared.

In parallel to a linear pattern of cellular injury as evident by constant rises of cTnI, myocardial contractility of the ischemic region remained significantly suppressed in LPS treated animals. This finding suggested that a significant myocardial stunning was still present although the resultant cell death was more moderated. These findings were in a weak agreement with previous studies that had observed beneficial effects of LPS pretreatment and its associated increases in TNFα levels on ischemic myocardium. Yao et al^12^ demonstrated that LPS pre-treatment reduced apoptosis of myocardial cells following an ischemia reperfusion injury and decreased MDA levels in the myocardium. Several other studies reported that LPS pre-treatment was associated with reduction in myocardial infarct size.^13-16^ Since different models of ischemia-reperfusion injury were used in these studies, no direct correlation could be concluded with our findings. We further showed that higher doses of LPS were not associated with improvement in cardiac function, and in addition, excessive doses of endotoxin had other deleterious effects on contractility and relaxation of myocardium.

Since soluble TNFαR also blocked the LPS-induced changes, these new findings appeared to result from LPS-induced generation of TNFα, which rose linearly until a plateau was reached. Effects of TNFα are induced by its binding to 2 different receptors: TNFα receptor type 1 and type 2. Both receptor types are found in the myocardium and activated via trimerization upon TNFα binding. In addition, it could potentially prevent NF-kB linked anti-apoptotic pathways, which may protect the heart during reperfusion.^17-20^ Our findings confirmed that following administration of soluble TNFαR, ischemic cellular injury was decreased, but ischemic impairment of myocardial contractility remained unchanged. These findings clearly demonstrated that detrimental effects of LPS on ischemia-reperfusion induced insult were mediated through the action of TNFα on the myocardium. One unique characteristic of this study was its graded action of LPS-induced increases in TNFα. Additionally, Brown et al demonstrated a decrease in myocardial injury of ischemia-reperfusion by TNFα pre-treatment.^21^ Even though the direct administration of TNFα was not used in these experiments, other studies clearly demonstrated a strong association of this cytokine in mediating the actions of LPS.^22,23^ Evaluation of LPS-induced TNFα levels showed a dose-dependent changes in cardiac function (myocardial contractility and relaxation), as well as local inflammatory response and myocardial injury. Global function of the heart (both contraction and relaxation) was only minimally deteriorated with lower serum TNFα concentrations. This observation was consistent with the previously demonstrated protective effects of this cytokine on mitochondria of the ischemic myocardium described by another study.^24^

Superiority of the sTNFαR-treated experiments in cardio-protection was likely due to its multiple targeted ligand binding. We also demonstrated that soluble TNFαR administration was associated with decreased levels of the biomarkers of myocardial injury, MDA and serum cTnI. Nevertheless, the ventricular segment shortening was not altered in either ischemic or non-ischemic regions following sTNFαR administration. Indeed, as anticipated, sTNFαR administration had no effect on LPS-induced increases of serum TNFα following myocardial ischemia, because this drug’s effects were merely post-translational. It is not clear yet why sTNFαR could have decreased ischemic cellular injury but had no effect on contractility. However, preservation of cardiac myocytes in the absence of functional improvement clearly indicated a significant ongoing myocardial stunning.

One of the main limitations of this study was to use endotoxin injection as one arm of the double-hit model that mimicked systemic sepsis. Although this study sheds light into the role of TLR-4 cytokines in mediation of ischemic injury, LPS administration is not a true reflection of septic shock both in animal models and clinical settings. Animal models of septic shock such as cecal ligation may be used to address this issue. These models will obviously involve a combination of cytokine and cellular inflammatory responses when compared to more targeted cascade of TNFα in current study.

In summary, we present a double-hit model, which employs the insults of endotoxemia with myocardial ischemia, ie, a combination that is not very uncommon clinical condition in critical care settings. A clear understanding of the underlying pathophysiology enables clinicians to develop therapeutic strategies that may improve the clinical outcome. Our findings clearly support the conclusion that while exuberant cytokine may be detrimental to myocardial well being during an additional ischemic insult, more moderated levels of these cytokines may modulate cardiac cell survival after an ischemic insult during systemic endotoxemia. These results may also help explain the previously disappointing clinical results that were obtained from treating patients with septic shock with anti-TNFα antibodies. A new approach to therapy of these combined insults may include moderation rather than elimination of TNFα bioactivity. However, more studies are needed to address the potential benefits of immunomodulation (cytokine suppression) as possible therapeutic strategies to these clinical pathologies in animal models prior to being tested in humans.

## Acknowledgments

Financial support used for the study was provided from a grant-in-aid awarded to NDN by the American Heart Association. (AHA 0060430Z).

## Ethical issues

None.

## Competing interests

None.

## References

[1] Rudiger A, Singer M (2007). Mechanisms of sepsis-induced cardiac dysfunction. Crit Care Med.

[2] Poelaert J, Declerck C, Vogelaers D, Colardyn F, Visser CA (1997). Left ventricular systolic and diastolic function in septic shock. Intensive Care Med.

[3] Levy RJ, Piel DA, Acton PD, Zhou R, Ferrari VA, Karp JS (2005). Evidence of myocardial hibernation in the septic heart. Crit Care Med.

[4] Heusch G (2013). Cardioprotection: chances and challenges of its translation to the clinic. Lancet.

[5] Calabresi L, Rossoni G, Gomaraschi M, Sisto F, Berti F, Franceschini G (2003). High-density lipoproteins protect isolated rat hearts from ischemia-reperfusion injury by reducing cardiac tumor necrosis factor-alpha content and enhancing prostaglandin release. Circ Res.

[6] Johansen D, Ytrehus K, Baxter GF (2006). Exogenous hydrogen sulfide (H2S) protects against regional myocardial ischemia-reperfusion injury--Evidence for a role of K ATP channels. Basic Res Cardiol.

[7] Xu Y, Arenas IA, Armstrong SJ, Plahta WC, Xu H, Davidge ST (2006). Estrogen improves cardiac recovery after ischemia/reperfusion by decreasing tumor necrosis factor-alpha. Cardiovasc Res.

[8] Asgeri M, Pourafkari L, Kundra A, Javadzadegan H, Negargar S, Nader ND (2014). Dual effects of tumor necrosis factor alpha on myocardial injury following prolonged hypoperfusion of the heart. Immunol Invest.

[9] Nader ND, Li CM, Khadra WZ, Reedy R, Panos AL (2004). Anesthetic myocardial protection with sevoflurane. J Cardiothorac Vasc Anesth.

[10] Nader ND, McQuiller PS, Raghavendran K, Spengler RN, Knight PR (2007). The role of alveolar macrophages in the pathogenesis of aspiration pneumonitis. Immunol Invest.

[11] Kawaguchi A, Nakaya H, Okabe T (2009). Blocking of tumor necrosis factor activity promotes natural repair of osteochondral defects in rabbit knee. Acta Orthop.

[12] Belosjorow S, Schulz R, Dorge H, Schade FU, Heusch G (1999). Endotoxin and ischemic preconditioning: TNF-alpha concentration and myocardial infarct development in rabbits. Am J Physiol.

[13] Hiasa G, Hamada M, Ikeda S, Hiwada K (2001). Ischemic preconditioning and lipopolysaccharide attenuate nuclear factor-kappaB activation and gene expression of inflammatory cytokines in the ischemia-reperfused rat heart. Jpn Circ J.

[14] Shames BD, Barton HH, Reznikov LL, Cairns CB, Banerjee A, Harken AH (2002). Ischemia alone is sufficient to induce TNF-alpha mRNA and peptide in the myocardium. Shock.

[15] Sivarajah A, McDonald MC, Thiemermann C (2005). The cardioprotective effects of preconditioning with endotoxin, but not ischemia, are abolished by a peroxisome proliferator-activated receptor-gamma antagonist. J Pharmacol Exp Ther.

[16] Zacharowski K, Frank S, Otto M, Chatterjee PK, Cuzzocrea S, Hafner G, Pfeilschifter J (2000). Lipoteichoic acid induces delayed protection in the rat heart: A comparison with endotoxin. Arteriosclerosis, Thrombosis & Vascular Biology.

[17] Aggarwal BB, Shishodia S, Takada Y (2006). TNF blockade: an inflammatory issue. Ernst Schering Res Found Workshop.

[18] Kubota T, Miyagishima M, Frye CS, Alber SM, Bounoutas GS, Kadokami T (2001). Overexpression of tumor necrosis factor- alpha activates both anti- and pro-apoptotic pathways in the myocardium. J Mol Cell Cardiol.

[19] Regula KM, Baetz D, Kirshenbaum LA (2004). Nuclear factor-kappaB represses hypoxia-induced mitochondrial defects and cell death of ventricular myocytes. Circulation.

[20] Valen G, Yan ZQ, Hansson GK (2001). Nuclear factor kappa-B and the heart. J Am Coll Cardiol.

[21] Brown JM, Anderson BO, Repine JE, Shanley PF, White CW, Grosso MA (1992). Neutrophils contribute to TNF induced myocardial tolerance to ischaemia. Journal of Molecular & Cellular Cardiology.

[22] Gu Q, Yang XP, Bonde P, DiPaula A, Fox-Talbot K, Becker LC (2006). Inhibition of TNF-alpha reduces myocardial injury and proinflammatory pathways following ischemia-reperfusion in the dog. J Cardiovasc Pharmacol.

[23] Xiao H, Liao YH, Chen ZJ (2008). Tumor necrosis factor-alpha: a new mechanism of ischemic ventricular fibrillation?. Chin Med J (Engl).

[24] Lacerda L, McCarthy J, Mungly SF, Lynn EG, Sack MN, Opie LH (2010). TNFalpha protects cardiac mitochondria independently of its cell surface receptors. Basic Res Cardiol.

